# Does access through the pronator quadratus influence pronation strength in palmar plate fixation of distal radius fractures in elderly patients?

**DOI:** 10.1007/s00402-023-04847-5

**Published:** 2023-03-27

**Authors:** Steffi S. I. Falk, Anna Maksimow, Thomas Mittlmeier, Georg Gradl

**Affiliations:** 1 Clinic of Trauma, Hand and Reconstructive Surgery, University of Rostock, Schillingallee 35, 18055 Rostock, Germany; 2 Clinic of Trauma, Orthopaedic, Hand and Reconstructive Surgery, Munich Municipal Hospital Group, Clinic Harlaching, Sanatoriumsplatz 2, 81545 Munich, Germany

**Keywords:** Distal radius fracture, Geriatric patients, Elderly, Pronation strength, Musculus quadratus pronator

## Abstract

**Introduction:**

Palmar plate fixation of the distal radius fracture involves dissecting the pronator quadratus (PQ). This is regardless of whether the approach is radial or ulnar to the flexor carpi radialis (FCR) tendon. It is not yet clear whether and to what extent this dissection leads to a functional loss of pronation or pronation strength. The aim of this study was to investigate the functional recovery of pronation and pronation strength after dissection of the PQ without suturing.

**Materials and methods:**

From October 2010 to November 2011, patients aged over 65 with fracture were prospectively enrolled in this study. Fracture stabilisation was performed via the FCR approach without suturing the PQ. Follow-up examinations took place 8 weeks and 12 months postoperatively, and pronation and supination strength were analysed by means of an especially developed measuring device.

**Results:**

212 patients were initially screened and 107 were enrolled. The range of motion compared to the healthy opposite side was Ext/Flex 75/66% 8 weeks postoperatively. Pronation was 97% with a pronation strength of 59%. After 1 year, the scores improved to Ext/Flex 83/80%. Pronation recovered to 99% and pronation strength to 78%.

**Conclusion:**

The present study can show a recovery of pronation as well as pronation strength in a large patient population. At the same time, the pronation strength is still significantly lower 1 year after the operation than on the opposing healthy side. As the pronation strength recovers as the grip strength and is at all times on a par with the supination strength, we believe that we can continue to refrain from re-fixating the pronator quadratus.

## Introduction

The radius fracture is the most common human bone fracture, and may especially be considered the fracture of the elderly. At up to 12:1,000, it has the highest incidence in the age group from 65 to 85 years [1–5]. Among the population group of over 60 years of age, which still completely cares for itself, 30–60% of the individuals fall once a year, and half of them even more often. About 6% of these falls result in bone fractures [6] of which the distal radius fracture is the most common [7]. In consideration of these statistics and the ongoing demographic change, it is obvious that good care of distal radius fractures is a prerequisite for the continuation of a self-determined and independent life. Therefore, a stable early functional fracture treatment is necessary in this section of the population.

The literature knows a wide variety of treatment options for distal radius fractures, such as conservative plaster treatment or reduction and stabilisation with K-wires, locking plates, nail or external fixator variants. Following response to the work of Altisimmi et al. [8] as well as Cooney and colleagues [9] and the increasingly surgical management of distal radius fractures, the majority are currently fixed using locking plates [10–12]. This usually allows immediate release for functional postoperative treatment without weightbearing.

On the one hand, the elderly in particular benefit from an early release of mobility of the wrist to maintain their current living conditions; on the other hand, they present with comorbidities that increase the risk of surgery. Thanks to regional anaesthesia, surgery is nowadays also possible for multimorbid patients. Since the introduction of locking plates, the palmar position has been favoured, if possible, between the two approaches for plate insertion, palmar and dorsal, respectively [13]. The surgical approach for palmar angular stable plate fixation is either radial to the flexor carpi radialis tendon or ulnar to it. Regardless of the choice of approach, exposure of the distal radius involves incision and release of the pronator quadratus (PQ). With this technique, the muscle most important for pronation [14] loses its function, at least temporarily.

With the increasing use of the palmar approach to the radius, a discussion was initiated about the handling of the PQ after plate osteosynthesis and the suture reconstruction of the muscle is discussed controversially. For example, Ateschrang et al. see evidence of an increased risk of tendon irritation without covering the plate with PQ and therefore favour suturing [11]. There is also work describing a minimally invasive partial release of the muscle for the preparation of the plate layer to prevent functional limitation and tendon irritation. In small groups of patients, however, the advantage of the minimally invasive release over the suture technique has not been convincingly demonstrated [15]. These controversial observations raise the question of whether the advantage of good soft-tissue coverage through palmar insertion of the plate comes at the cost of functionality due to the loss of the PQ, if intraoperative suturing is not performed.

As there are very little data in the literature, especially on pronation strength, we decided to perform a post hoc analysis of our unpublished data from a prospective study of patients with distal radius fractures in which pronation and supination strength were measured. To our knowledge, this is the first study to investigate the recovery of pronation and pronation strength over 12 months in elderly patients after non-suture of the PQ.

## Method

The primary prospective study included patients who underwent surgery between October 2010 and November 2011. For this post hoc analysis, we enrolled patients who were over 65 years old and had been treated with a palmar plate for a distal radius fracture. No patients with bilateral fractures or a fracture of the contralateral wrist within the last 24 months were included. Fracture classification was performed according to the criteria of the AO foundation valid in 2010. Patients with type B fractures were excluded due to the small number of cases in the study period.

The fractures were stabilised via the Henry approach using the Optimus locking plate (MORE Medical, Germany). The radius fracture was treated under general or regional anaesthesia. Re-fixation of the PQ was generally not performed. All patients were treated with partial weightbearing for 6 weeks and early functional posttreatment with free mobility 7 days after surgery. Clinical and radiological follow-up examinations took place after 8 weeks and 12 months, respectively.

**Fig. 1:**
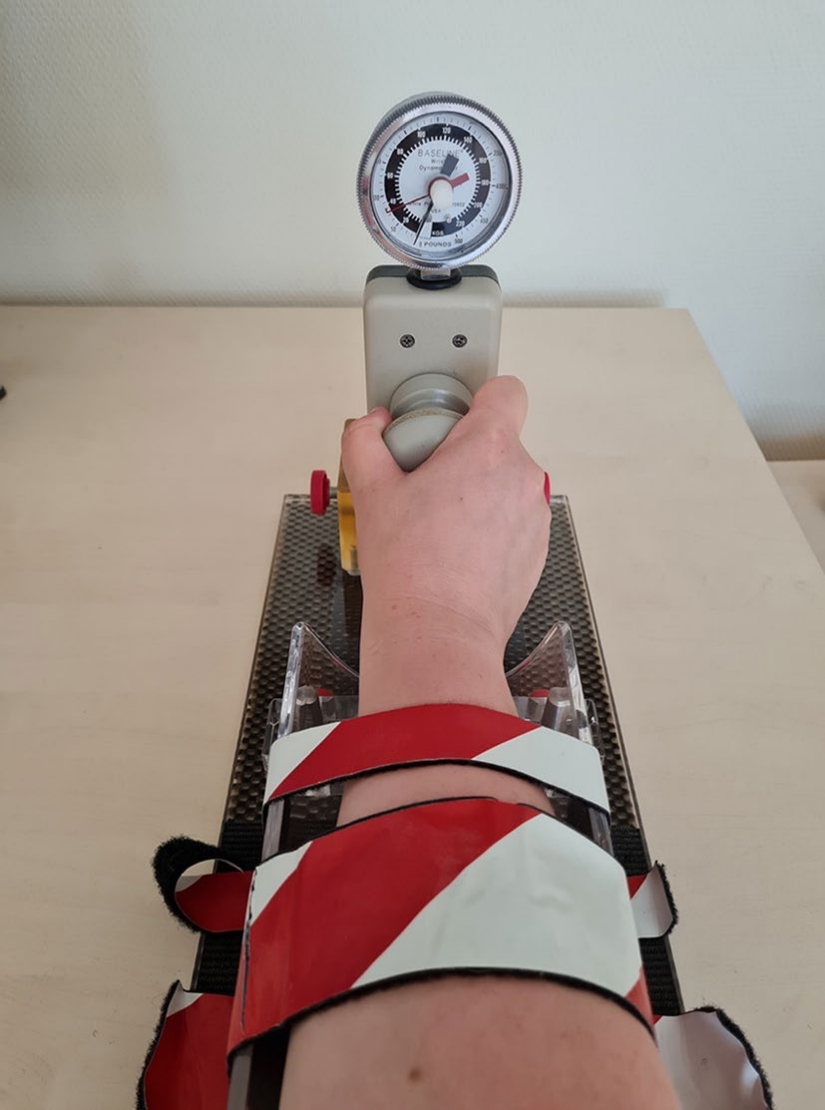
The Baseline Wrist Dynamometer (Fabrication Enterprises, USA) for measuring pronation and supination force as used in the patient examination

The follow-up examinations included the measurement of all ranges of motion according to the neutral zero method using a goniometer. Grip strength was assessed in all patients as the maximum of three measurements on the Jamar dynamometer. The Baseline Wrist Dynamometer (Fabrication Enterprises, USA) with a knob, an especially developed device for measurement of pronation and supination forces, was used to determine pronation force (Fig. 1).

The gold standard of strength measurement is represented by work simulators and isokinetic rehabilitation and measurement devices, such as the Cybex 6000 [16]. However, these devices are large and cost-intensive, so a handy and easy-to-use device had to be found without sacrificing accurate and reliable measurement results. The Baseline Hydraulic Dynamometer (Fabrication Enterprises, USA) was compared in a study with the gold standard for measuring pronation and supination force, the Cybex 6000, and found to be a portable, reliable and valid tool for measuring pronation strength when equipped with a door knob handle. A holder with a support plate for the arm to be measured was made especially for the Baseline Wrist Dynamometer at our hospital. We used the holder presented by Wong et al. as a basis [16]. The holder is intended to prevent positional changes, such as the unconscious lifting of the arm, which would lead to the activation of other muscles responsible for pronation. All patients were allowed to practice turning the door knob in a first step, and then, after a short relaxation period, the measurements were taken.

The values for movement and force were calculated as a percentage compared to the unaffected wrist. The follow-up examination also included the assessment of pain at rest and during movement using the Visual Analogue Scale (0–10), the determination of the Gartland and Werley score (0–34) [17] and the Castaing score (0–27) [18]. Both scores were developed for wrist fractures and assign points for, e.g., deformity, loss of function and pain, i.e., they range from zero points for an excellent result to 20 or 25 points and more for a poor result.

The statistical analysis was carried out using SPSS version 28. Descriptive data analyses and analyses for normal distribution were done using the Kolmogorov–Smirnov test. In the case of predominantly non-normally distributed variables, the comparison between the fracture types was carried out using the Mann–Whitney *U* test. The significance level was set at *p* = 0.05. The correlation calculations were carried out according to Spearman.

## Results

**Fig. 2:**
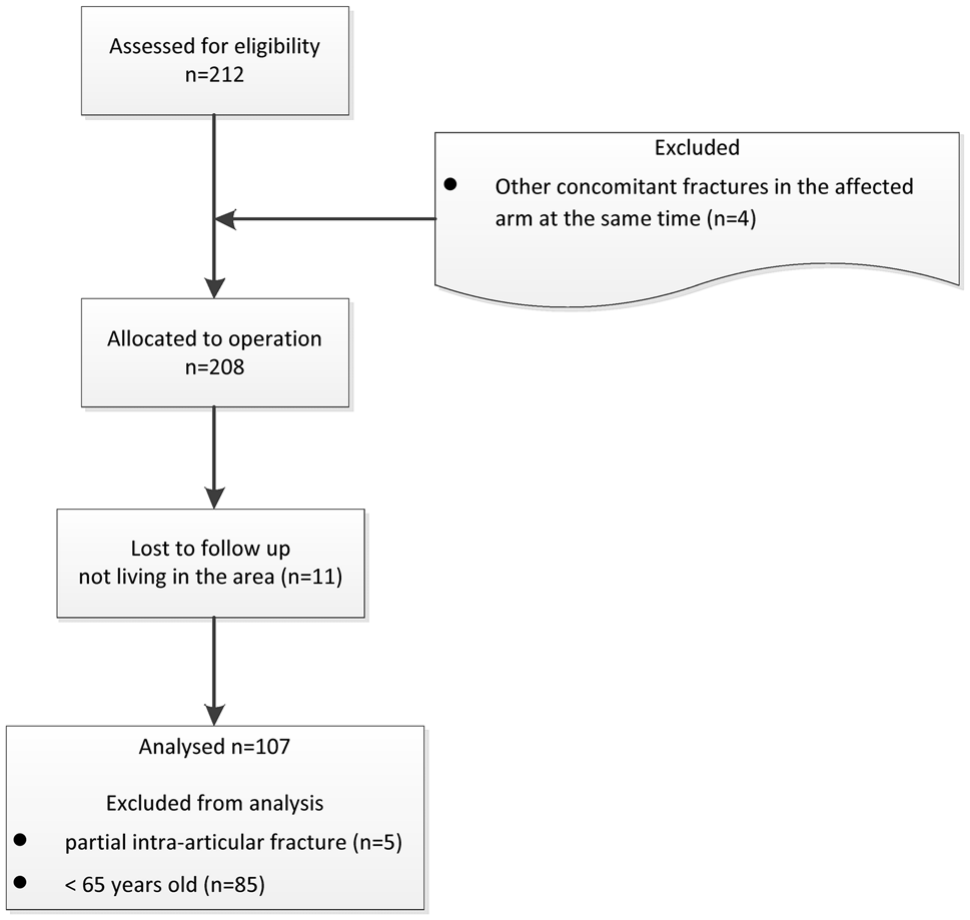
Flow diagram

All data are given as mean values with the standard deviation in brackets. Of the 212 patients screened, 107 could be included in this study (see Fig. 2). The patients had an average age of 74.55 (6.54) years. According to the inclusion criteria, the youngest patient was 65 years old and the oldest 92 years. The proportion of men was 6.5% for the entire patient population. This resulted in a gender distribution of about 1:15.

**Fig. 3:**
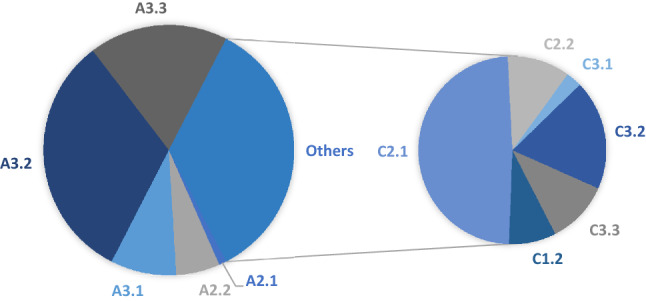
Distribution of fracture types according to the classification of the AO Foundation which was valid in 2010

According to the AO classification, 65% of the patients suffered from a type A fracture and 35% of a type C fracture (see Fig. 3). As there were statistically significant differences between the two fracture groups with regard to pronation strength after 8 weeks, the patients were analysed depending on their type of fracture and not combined into one large group.

**Fig. 4:**
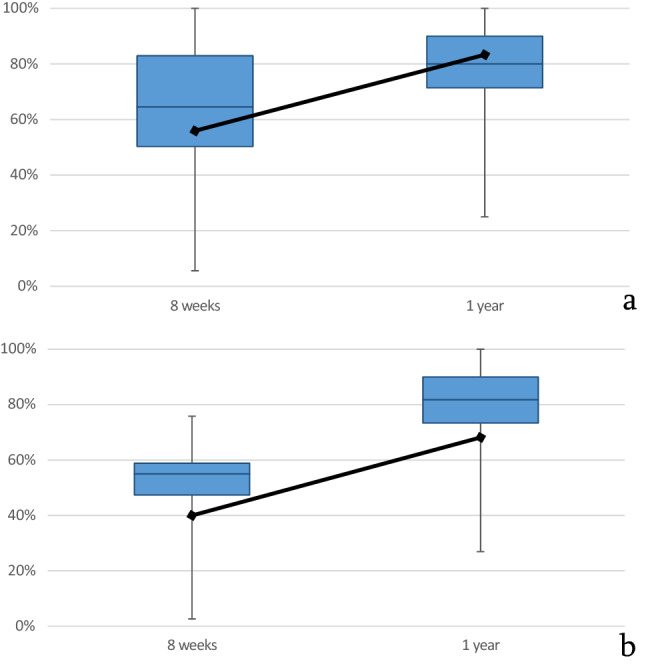
Pronation strength in percent to the healthy side compared at 8 weeks and 1 year. The median grip strength for the same period is also shown as a black line: a for type A fractures and b for type C fractures

The range of motion in percent to the healthy side was pro/sup 97/96% after 8 weeks (Table 1). The pronation strength was already 54% and 65%. The Gartland and Werley score was already good with an average of eight points. In the radiological examinations, all fractures were consolidated at eight weeks. After 1 year, all scores improved, including the pro/sup to 99/98% (see Table 2). The pronation strength recovered to 78% (Fig. 4). The Gartland and Werley score remained good at four points after 1 year and the pain almost completely decreased with a VAS score of two under motion.

**Table 1:** Statistical analysis of the 8-week examination values separated according to A and C fracture as mean value with the standard deviation in brackets

	Type A fractures	Type C fractures	*P* value
Age	74.89 (6.22)	73.75 (5.09)	0.615
Time period	9.33 (1.68)	9.79 (1.38)	0.074
Pronation/supination characteristics			
Pronation	97.04 (6.48)	96.08 (5.47)	0.336
Supination	90.56 (19.53)	96.08 (5.47)	0.579
Pronation strength	65.31 (22.13)	53.86 (13.13)	**0.030**
Supination strength	61.39 (24.11)	54.92 (17.00)	0.254
Further characteristics			
Extension	74.42 (24.1)	75.56 (18.9)	0.956
Flexion	68.44 (17.67)	64.43 (21.11)	0.682
Radial abduction	73.54 (25.17)	75.74 (27.66)	0.554
Ulnar abduction	71.85 (21.2)	68.29 (19.61)	0.329
Grip strength	50.83 (22.81)	42.87 (12.35)	0.207
VAS at rest	1.47 (2.45)	2.35 (2.55)	0.131
VAS at movement	3.57 (2.88)	4.88 (2.45)	0.181
Casting score	5.79 (4.35)	7.88 (3.69)	0.075
Gartland score	7.00 (4.28)	9.18 (4.57)	0.129

Significant *P* value is in bold

All movement measurements are given in percent of contralateral intact side. The time period is given here in weeks

**Table 2:** Statistical analysis of the 1-year examination values separated according to type A and C fractures as mean value with the standard deviation in brackets

	Type A fractures	Type C fractures	*P* value
Time period	16.47 (4.12)	15.93 (2.28)	0.963
Pronation/supination characteristics			
Pronation	98.67 (3.69)	99.15 (3.08)	0.832
Supination	97.33 (5.81)	98.29 (6.16)	0.584
Pronation strength	76.80 (21.12)	80.37 (13.12)	0.808
Supination strength	75.82 (21.36)	81.57 (15.96)	0.564
Further characteristics			
Extension	84.68 (17.28)	82.48 (16.26)	0.484
Flexion	80.35 (18.05)	79.05 (19.78)	0.903
Radial abduction	73.64 (19.07)	87.28 (13.66)	**0.038**
Ulnar abduction	84.92 (16.55)	87.43 (16.05)	0.447
Grip strength	77.62 (23.43)	69.67 (25.12)	0.345
VAS at rest	1.04 (2.11)	0.85 (2.15)	0.716
VAS at movement	2.36 (2.78)	2.38 (2.29)	0.785
Casting score	3.00 (3.11)	4.15 (3.46)	0.210
Gartland score	3.88 (3.57)	4.62 (4.41)	0.761

Significant *P* value is in bold

All movement measurements are given in percent of contralateral intact side. The time period is given in months

In terms of improvement in movement, despite very good range of motion, pronation and supination also recovered to normal values with at least 97% of the opposite side. Both pronation and supination strength were also able to increase significantly. Both strength values ultimately recovered in the same range as the grip strength and reached a value of at least 71% of the healthy side. It was noticeable that the grip strength for both types of fracture was significantly lower than the pronation strength after eight weeks. For extra-articular fractures, both forces reached approximately the same level after 1 year, while for intra-articular fractures, the grip strength remained below the pronation strength. The detailed development of the values is shown in Table 3.

**Table 3:** Overview of the improvement of the wrist function for the turning movement between both examinations

	At 8 weeks	At 1 year
*Type A fractures*		
Pronation	97.04 (6.48)	98.67 (3.69)
Supination	90.56 (19.53)	97.33 (5.81)
Pronation strength	65.31 (22.13)	76.80 (21.12)
Supination strength	61.39 (24.11)	75.82 (21.36)
Grip strength	50.83 (22.81)	77.62 (23.43)
*Type C fractures*		
Pronation	96.08 (5.47)	99.15 (3.08)
Supination	96.08 (5.47)	98.29 (6.16)
Pronation strength	53.86 (13.13)	80.37 (13.12)
Supination strength	54.92 (17.00)	81.57 (15.96)
Grip strength	42.87 (12.35)	69.67 (25.12)

Within 12 months after surgery, four patients underwent implant removal. All four implant removals were done at the patient's request. Tendon irritation or rupture did not occur in either group of patients.

**Fig. 5:**
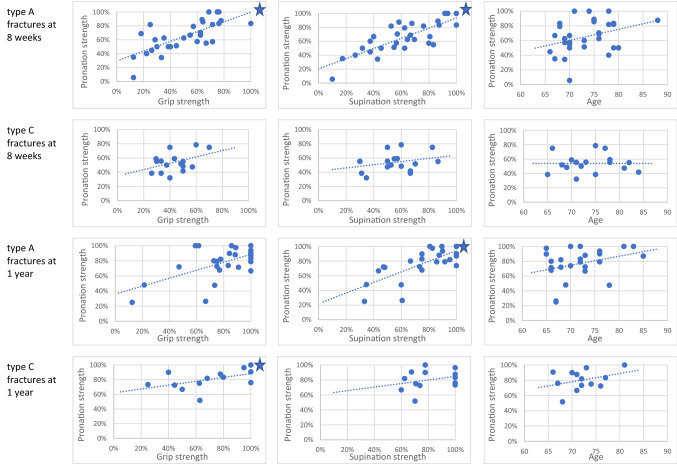
Scatter diagram of the correlations divided according to the time of examination and the fracture type. Each diagram was supplemented with a trend line for a better overview. Significant correlation are marked with a star in the right upper corner

The analysis of correlations between pronation strength and pronation, supination strength as well as grip strength and age showed strong correlations for the examination after 8 weeks, which weakened considerably for the examination after 1 year. It is also striking that these correlations could be proven for type A fractures, but hardly for type C fractures. Figure 5 contains the scatter diagrams for the investigated characteristics.

For pronation, extra-articular fractures showed a slightly positive correlation at the 8-week examination (*k* = 0.433, *p* = 0.017), which could no longer be proven after 1 year (*k* = 0.111, *p* = 0.597). For intra-articular fractures, there was a slight negative correlation after 8 weeks (*k* = − 0.302, *p* = 0.239), which could also be detected at the final examination (*k* = − 0.386, *p* = 0.193).

The correlation to the supination strength was almost constantly strong in both examinations of the extra-articular fractures with *k* = 0.757 (*p* = 0.001) and *k* = 0.722 (p = 0.001). This strong correlation was not as evident in the patients with intra-articular fractures. At the 8-week examination, there was no correlation with k = 0.117 (*p* = 0.656) and after 1 year with *k* = 0.342 (*p* = 0.252) a minimal not significant correlation.

While the correlation of pronation strength to grip strength was still very clear at k = 0.727 (*p* = 0.001) after 8 weeks for extra-articular fractures, a correlation coefficient of only *k* = 0.373 (*p* = 0.066) was shown after 1 year. While this correlation was still missing for intra-articular fractures at the first examination (*k* = 0.196, *p* = 0.451), it can also be proven at the 1-year follow-up examination with *k* = 0.597 (*p* = 0.031).

Age showed a minimal correlation for extra-articular fractures with k = 0.324 (*p* = 0.08) in the examination after 8 weeks, which held at *k* = 0.294 (*p* = 0.154) after 1 year. For intra-articular fractures, the correlation coefficient was not greater than *k* = 0.154 (*p* = 0.615) at any time.

## Discussion

Among the multitude of therapeutic options after distal radius fractures, palmar locking plate fixation has increased in importance and is currently a common standard procedure even for elderly patients [13]. The advantage of palmar plating is the good soft-tissue coverage and the lower complication rate [19]. At the same time, this therapy also means that the PQ is dissected longitudinally to expose the fracture. This has to be done regardless of whether the approach is radial or ulnar to the flexor carpi radialis tendon. Thus, the strongest pronator in the wrist becomes functionless in the short term [14]. Until now, it was unclear to what extent this muscle dissection leads to functional losses in pronation function or pronation strength development. The present study was able to show a transient loss of function in the early postoperative period but also a recovery in a large group of patients after 1 year.

In the literature, the repair of the PQ or its suture is controversially discussed. The basic idea for this was described by Ateschrang et al. [11] as tendon irritation of the flexors due to lack of soft-tissue coverage of the plate by the PQ. Therefore, this study group favours suturing the muscle to prevent tendon irritation and the risk of rupture. Brown et al. [20] also recommend suturing the PQ, while noting, nevertheless, that tendon irritation can occur. In addition to suturing, PQ-sparing procedures have also been proposed in the other publications. The latter methods often involve tunneling through periosteal release, so that the plate can then be inserted [15, 21]. This procedure, however, does not allow exposure of the fracture gap and reduction under visual control, which many surgeons do not want to miss. These procedures have therefore not yet become widely accepted. In contrast, various suture techniques for re-fixation of the PQ are described in the literature. These include the simple interrupted suture, the braided suture, the suture combined with prolonged immobilisation or the suture of the PQ together with connective tissue fibres from the first extensor tendon compartment or the palmar limb of the brachioradialis tendon [22, 23].

Besides the consideration of preventing tendon irritation, the question of functional recovery then came to the fore. Thus, Hershman et al. compared functional and subjective parameters in groups with and without re-fixation of the PQ and concluded that suturing the PQ had no influence on clinical outcome. Wrist movement and patient satisfaction did not differ in the groups studied. Instead, the group with PQ repair had a higher rate of re-operations with implant removals. This even led to the thesis that the suture is more likely to cause problems, because it entails additional thickness to the plate [24]. Similarly, Goorens et al. examined wrist function and strength in patients with and without PQ sutures. They showed that grip strength was better at 6 weeks and that there were no differences in function at the 6-month follow-up. They also emphasise that the PQ has a minimal effect on wrist strength due to its function/biomechanics. The research group did not measure pronation strength to clarify clinical functional recovery beyond range of motion [25]. Fang et al. studied the healing of PQ after suturing. They reported only 23 healed PQ with concomitant muscle atrophy in 126 patients after suturing, with the remaining patients showing scarring intraoperatively. Healing was assessed at implant removal by experienced surgeons [26]. The research group of Mulders et al. investigated the question of functional differences between sutured and non-sutured PQ in a systematic review. After an initial identification of 320 trials, only two studies remained for evaluation. Both did not take pronation strength into account. Therefore, the authors conclude that there are still too little data to answer this question and they critically question the quality of PQ sutures [27]. Maniglio et al. investigated the influence of the PQ on wrist function and wrist strength in a cadaver study to determine the need for PQ repair. They found no difference in wrist strength, but like most clinical trials, they did not examine pronation strength [28].

Currently, only a few studies with small sample sizes exist on the effect on postoperative pronation strength [29]. For example, Hohendorff et al. [30] promoted a repair method using the brachioradialis that achieved good soft-tissue coverage, but no better function in pronation or pronation strength. Häberle et al.’s [31] study of suturing the PQ in a very small study group showed a benefit in postoperative pain but also no improvement in pronation or pronation strength. Huh et al. [32] and Chirpaz-Cerbat [33] also studied the pronation strength of their patients after suturing and could not find any significant differences between the sutured and the non-operated side after 1 year.

Goorens et al. [15] and Huang et al. [34] went into further detail comparing pronation motion between the surgical technique of suturing the PQ and an initial minimally invasive approach through the PQ. No difference in pronation was to be shown between the groups. Both groups of authors advocated the minimally invasive approach and justified this by less postoperative pain and intraoperative blood loss. Goorens et al. [15] also showed an improvement in flexion and extension in the minimally invasive approach group in their study. Both did not examine pronation strength in the follow-up studies. Furthermore, all these studies were not focused on exclusively elderly patients. In none of these studies mentioned, the average patient age was over a mean of 61 years.

A strength of the prospective study presented here is, therefore, that it provided new data on objective pronation strength measurements in the group of elderly patients. With an average age of 74 years, the patient sample considered corresponds to the advanced age expected of an elderly population. The distribution of women and men in this evaluation was higher in favour of women than generally described in the literature. The studies on incidences and gender distributions in distal radius fractures by Jerrhag et al. [35] were able to show that the gender ratio changed in favour of women with increasing age. It should be considered that these figures apply to the overall cohort of fractures and not to the subgroup of those treated surgically. In the study of surgically treated patients over 50 years of age by Sirniö et al. [36], the ratio of women to men was 20:1. From this point of view, the gender ratio evaluated here corresponded to the observed ratio.

The motion ranges were given as a percentage of the healthy side [21, 37, 38]. This specification takes into account a decay of muscle function and strength limitation with age. Normalisation to the opposite side eliminates any bias due to interindividual as well as gender differences in range of motion or strength and makes individual patients comparable to each other. The Baseline Hydraulic Dynamometer (Fabrication Enterprises, USA) used in this study had been compared to a gold standard for measuring pronation strength, the Cybex 6000 dynamometer, and was found to represent a reliable and valid tool for measuring pronation strength when used with a doorknob or handle, as in the study presented here [16].

The results of the range of motion after 8 weeks were already in a good range for the patient with a minimum range of 66% of the opposite side. With 96% after 8 weeks, pronation was almost in the range of the healthy side and, thus, was barely affected by the muscle dissection. At this time, the grip strength was still limited to slightly less than 50% of the opposite side. For us, this resulted from the fact that strength could not be trained for six weeks in contrast to movement, which was treated immediately functionally. Pronation strength seemed to be less affected by the lack of strength exercise. The pronation strength had already recovered to an average of 59% at the 8-week follow-up. The study by Chirpaz-Cerbat [33] showed a recovery of pronation strength to not quite 40% and grip strength to slightly more than 50% of the healthy opposite side after 6 weeks in a significantly younger patient group after suturing the PQ. These results after suturing are in the range of the functional recovery also achieved in this study.

After 1 year, the pronation strength had improved by 19% to 78%. With this value, the pronation strength was on par with the grip strength, which reached an average of 73%. Thus, the recorded pronation strength was lower than reported in the literature. Chirpaz-Cerbat [33] further reported a recovery of slightly over 80% of pronation and grip strength compared to the healthy opposite side. However, it must be considered that the patient group evaluated in their study was significantly younger at a mean of 48 years of age. Considering the longer convalescence time at an older age, we consider the values comparable and assume that a further recovery of our patients is conceivable.

It should also be noted that the pronation strength was still lower 1 year after surgery compared to the healthy side. This circumstance is moderated by the fact that the pronation strength after 1 year is within the range of the supination strength, as it was at the 8-week follow-up examination. This is despite the fact that supination was not impaired by any iatrogenic muscle injury. If the grip strength is also taken into account, there is even a tendency towards better pronation strength. It is, therefore, even possible that the loss of strength does not originate from the muscle dissection, but from the actual fracture itself.

It should also be noted in this context that Huh et al. [32] were able to show that limitations at this level of strength do not have any negative effect on wrist function and, consequently, on patient satisfaction. This good degree of wrist function was also confirmed in this study. Although pronation was already almost in the range of the healthy side at 96% after 8 weeks, a recovery to 99% at the annual examination was also observed in our study. The other ranges of motion reached at least 80% of the opposite side. With these functional values, the patients examined here were within the range of other studies on plate osteosynthesis for distal radius fractures. This suggests that the strength values determined are also comparable [11, 39].

The calculations of the correlation of the pronation strength showed a large and significant correlation to the opposing supination strength and a small one to the grip strength and pronation. Age showed no correlation which is certainly due to the fact of using percentages of recovery. We are not aware of any previous study that had scrutinized these correlations. This could be due to the fact that there are very few studies that have determined postoperative pro- and supination strength at all. Shi et al. [29] conducted a meta-analysis on the question of the need to repair the PQ and were able to include six studies for this purpose. Of these, only three studies had measured pronation force at all. None of these studies examined the correlation with grip strength. All studies addressed the question of whether suturing or even repairing the PQ tendon provided a benefit. The results were inconsistent, with one positive and one negative result related to suturing the PQ.

To decide in favour of or against suturing the PQ, the eventual complications associated with a PQ reconstructive or preserving technique must be considered. Apart from an ischaemic contracture due to a too tight suture and consecutively reduced range of motion of the wrist, a compartment syndrome of the PQ and an iatrogenic radial artery injury might result [40, 41]. The compartment syndrome follows from the fact that the PQ lies in its own compartment. If the plate is pushed under the muscle as part of a minimally invasive technique, the surrounding fascia is not opened. Therefore, an increase in pressure might occur as part of a closed trauma [12].

## Limitations

The study presented here is a single-arm study without a control group. At the first glance, a control group seems reasonable, but it raises a number of methodological concerns. A wide variety of repair techniques for PQ are described in the literature and there is no standardisation. Similarly, studies show that suturing is associated with good healing of the PQ in only a few cases. In this context, it is questionable whether comparing two groups (one with sutures and one without) really compares different situations. Therefore, this trial has only one arm, and the question to be answered is whether the clinical functional recovery is so limited that suturing the PQ needs to be considered at all. To answer this question, there is no need for a control group.

With a maximum follow-up of 1 year, this study is at the median of the follow-up periods of published studies on distal radius fracture therapy [42, 43]. The data presented here are not intended to represent a long-term study, however, with a 12 month follow-up feature a prolonged time period for elderly patients. Especially, in the group of older people, the time immediately after an injury or surgery is already important. Against the background of a limited life expectancy, this group of patients cannot and does not want to wait 2 years or more for good function.

Similarly, the number of participants, especially for a study in the elderly setting, is comparable to other published studies. In the meta-analysis conducted by Li et al. [43] on the therapy of radial fractures in the elderly group, the median total number of participants was 90 and thus just below the number of participants included here.

## Summary

Thus, the present study could demonstrate a good functional recovery of pronation strength as well as pronation itself without suturing the dissected PQ in elderly patients. The presented data clearly illustrate that pronation is restored after only 8 weeks and that pronation strength is questionably affected by muscle dissection. The pronation strength improved in approximately the same proportion as the supination strength and grip strength. This suggests that the muscle regenerates or that the remaining muscles adapt in such a way that they can take over the function of the PQ. Since supination is independent of pronator function, there is no need for additional suturing of the muscle when the surgical wound is closed. Whether the loss of pronation strength is due to muscle dissection or the fracture itself cannot be answered in the setting of the investigation.

## Data Availability

Data supporting the study results are available from the corresponding author upon reasonable request.
